# Supplementary material for: Splice-Junction-Based Mapping of Alternative Isoforms in the Human Proteome
Source: Cell Rep. Author manuscript; Available in PMC 2020 Jan 15. (PMC6961840; doi:10.1016/j.celrep.2019.11.026)
Supplement: 2 [file NIHMS1546469-supplement-2.zip › DF1/PXD010154/Liver/Liver_32_SIRT7_AGPEVAGGAAEGAPPPGTPPPLPGPAMPGPR.pdf]

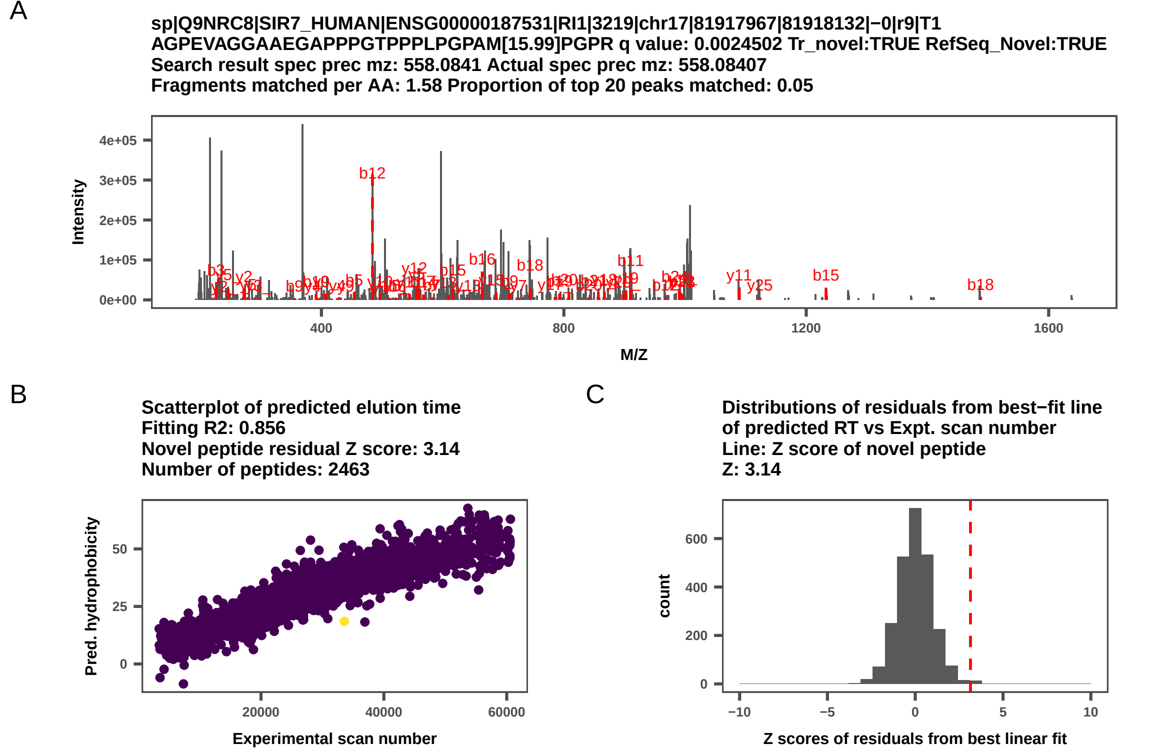
A
sp|Q9NRC8|SIR7_HUMAN|ENSG00000187531|RI1|3219|chr17|81917967|81918132|−0|r9|T1
AGPEVAGGAAEGAPPPGTPPPLPGPAM[15.99]PGPR q value: 0.0024502 Tr_novel:TRUE RefSeq_Novel:TRUE
Search result spec prec mz: 558.0841 Actual spec prec mz: 558.08407
Fragments matched per AA: 1.58 Proportion of top 20 peaks matched: 0.05
Intensity
4e+05
3e+05
2e+05
1e+05
0e+00
b12
b16
b18
b11
y11
y25
b15
b18
M/Z
400
800
1200
1600
B
Scatterplot of predicted elution time
Fitting R2: 0.856
Novel peptide residual Z score: 3.14
Number of peptides: 2463
Pred. hydrophobicity
50
25
0
20000
40000
60000
Experimental scan number
C
Distributions of residuals from best−fit line
of predicted RT vs Expt. scan number
Line: Z score of novel peptide
Z: 3.14
count
600
400
200
0
−10
−5
0
5
10
Z scores of residuals from best linear fit
